# Prevalence and determinants of physical violence against doctors in Bangladeshi tertiary care hospitals

**DOI:** 10.1186/s12960-023-00811-x

**Published:** 2023-03-28

**Authors:** Md. Shahjalal, Mohammad Morshad Alam, Md. Nafiul Alam Khan, Arifa Sultana, Sanjana Zaman, Ahmed Hossain, Mohammad Delwer Hossain Hawlader

**Affiliations:** 1 Department of Public Health, North South University, Dhaka, Bangladesh; 2 Research Rats, Dhaka, Bangladesh; 3 Global Health Institute, North South University, Dhaka, Bangladesh; 4 Statistics Department, Khulna University, Khulna, Bangladesh; 5 Department of Public Health, Daffodil International University, Dhaka, Bangladesh

**Keywords:** Physical violence, Doctor, Workplace violence, Hospital, Bangladesh

## Abstract

**Background:**

The increasing physical violence against doctors in the health sector has become an alarming global problem and a key concern for the health system in Bangladesh. This study aimed to determine the prevalence and associated factors of physical violence against doctors in Bangladeshi tertiary care hospitals.

**Methods:**

A cross-sectional survey was performed among 406 doctors working in tertiary care hospitals. Data were collected using a self-administered questionnaire and the binary logistic regression model was employed for predicting physical violence against doctors.

**Results:**

Of the participants, 50 (12.3%) doctors reported being exposed to physical violence in 12 months prior to the survey. According to logistic regression analysis, aged less than 30 years or younger, male and never-married doctors were prone to physical violence. Similarly, doctors from public hospitals and those worked in emergency departments were at higher risk of physical violence. More than 70% of victims reported that patients’ relatives were the main perpetrators. Two-thirds of the victims referred to violence in the hospitals as a grave concern.

**Conclusions:**

Physical violence against doctors is relatively common in the emergency departments and public hospitals in Bangladesh. This study found that male and younger doctors were at high risk of exposing physical violence. To prevent hospital violence, authorities must develop human resources, bolster patient protocol and offer physician training.

**Supplementary Information:**

The online version contains supplementary material available at 10.1186/s12960-023-00811-x.

## Introduction

Over the last decade, violence in healthcare systems showed an upward trend and continues to be a growing concern at the global level [1, 2]. It is estimated that more than half of all health workers have experienced violence at work in their careers due to direct contact with people [3]. According to the World Health Organization (WHO), about 8–38% of health workers are exposed to physical violence (including slapping, punching, kicking, stabs, shots, pushing, biting, pinching and murder) in their workplace [4]. In recent times, a rising number of incidents of physical violence against doctors has been reported and seems a common worldwide phenomenon [5–7].

A number of recent literatures have reported that physical violence is more prevalent among doctors and varies widely by countries. For example, 5.0% in the UK [8], 17.11% in Turkey [9], 19.08% in Syria [6], and 25. 4% in Pakistan [10]. In China, a study reported that 97% of violent hospital incidents were physical, and 81% of those incidents occurred against doctors from 2014 to 2018 [11].

A growing body of evidence indicated that the majority of physical violence incidents occurred in the hospital setting rather than outside [6, 8, 12]. The literature also documented that physical violence against doctors varies in departments and is disproportionately high in emergency departments [2, 13], surgery departments [14, 15] and psychiatric units [1, 16]. Several studies have recorded the experience of physical violence faced by doctors was more likely to take place against young doctors [10, 16], male doctors [5, 12], unmarried doctors [14, 17], and those working in public hospitals [6, 10]. Researchers also reported that physical violence against doctors was perpetrated mainly by patients [6, 16], patients’ relatives [6, 7], and hospital management [14, 16].

It is well established that health workers are particularly stressed because of their profession [1–4, 16–18], and doctors are at the top of that list [6–8, 14, 19]. As well it is also evident that physical violence is reported in many studies to have a lasting effect on these professionals’ productivity and mental health [6, 7, 18]. These incidents of violence may lead to doctors taking time off from work or even abandoning their profession which has serious repercussions on human resources for health in developing countries like Bangladesh, where the number of doctors is insufficient to meet the population’s needs [19–21]. Given the above context and concern about the rising threat of workplace violence in the health system, understanding the prevalence and associated factors of physical violence among doctors is critical for developing preventive strategies, achieving healthcare access for all and meeting the Sustainable Development Goals (SDGs) target in Bangladesh [22].

To date, to our knowledge, two studies were conducted among Bangladeshi doctors based on newspaper or media reports and compiled their experiences of violence on a social media platform called “Platform” between May 2014 and March 2018 [19, 23]. However, these studies were conducted without face-to-face interviews and overlooked the prevalence of physical violence, which highlights a critical research gap. Based on these limitations, we aimed to estimate the prevalence of physical violence against Bangladeshi doctors in tertiary care hospitals and its associated variables. With that, we also identified perpetrators’ characteristics and their consequences. Finally, we assessed participants' worrying levels due to physical violence and compiled potential suggestions from victims for tackling violence in hospitals.

## Methods

### Study design

This cross-sectional study was employed among doctors working in tertiary care hospitals in Bangladesh. The sample size was calculated based on the assumptions of an alpha of 0.05, a confidence interval (CI) of 95% and a 16% prevalence of physical violence from previous study [14]. The total calculated sample size for this study was 378. However, we collected data from 426 doctors for our study.

### Data collection

The study was conducted between April and June 2019. We used a self-administered questionnaire based on the Workplace Violence Survey Questionnaire (2003, ILO/ICN/WHO/PSI) [3] and the questionnaire used for this study is available in Additional file 1. The participants were conveniently invited from randomly selected seven tertiary care hospitals (Dhaka Medical College Hospital, Mugda Medical College Hospital, Holy Family Red Crescent Medical College Hospital, Shahabuddin Medical College Hospital, Pabna Medical College Hospital, Rangpur Medical College Hospital, and Chattagram International Medical College Hospital) in Bangladesh.

The data collection was done by the first author (MS) and a group of data collectors. All of them were doctors and/ or medical students. The questionnaire was provided to each participant during working hours and was collected with written consent. Before filling out the questionnaires, data collectors explained the purpose of the study and that it was confidential and voluntary. This study excluded doctors with less than one year of work experience in the hospital and was absent during data collection. Based on these exclusion criteria, approximately 500 doctors were asked to participate, while 436 doctors were interviewed (response rate was 87%). We collected 426 completed questionnaires and excluded data from 20 participants during validation process due to a lack of informative explanations. The final sample included 406 doctors for analytical exploration. The process of data collection, identification and inclusion flow diagram is presented in Additional file 2.

### Measures

#### Outcome measures

The main outcome was physical violence measured with a single self-reported question with two items with a response option of ‘yes’ or ‘no’: “During the past 12 months, did you experience physical violence at your working hospital?” The response was dichotomised (1 = yes; 0 = no). Physical violence was referred to physical action (e.g., beatings, kicks, slapping, stabbings, shootings, pushing, biting, and pinching) targeted against a person or group that results in physical harm or sexual harm [24].

#### Explanatory variables

##### Violence related factors

The perpetrators of violence were assessed by asking participants who the perpetrators were. The perpetrators of physical violence were considered with the questions: “Who was the aggressor toward you?” to which victims could response ‘patient’, ‘patient’s relatives’, and ‘visitors or unknown’. Injury and take off from work were assessed with the questions: “Were you injured as a result of violence”? and “Did you absent from your work after being a victim?” These responses were coded as ‘yes’ or ‘no’. Consequences for the perpetrator were assessed with the question: “What were the consequences of the perpetrator?” to which victims could response ‘none’, ‘verbal warning’, ‘care discontinued’, ‘reported to police’, and ‘don’t know’.

##### Psychological factor

A psychological factor included in this study was anxiety. Participants’ worried levels were assessed using the question: “How worried are you about your current workplace? The responses were coded as ‘0 = not at all’, ‘1 = a little worried’, ‘2 = worried’, and ‘3 = very worried’.

##### Preventive factors

Preventive factors included a question on suggested measures to tackle violence in the hospital. The question asked participants about proposed preventive measures in the hospitals. Participants were asked: “To what extent do you think these measures would be helpful in your work hospital?” to which participants could respond ‘increase doctors’, ‘maintain patient protocols’, ‘reduce working alone’, and ‘offer training or workshop’.

##### Socio-demographic factors

According to the majority of studies, the risk of violence is linked to gender, age, marital status, type of hospital, working department, and employment category. Therefore, these factors were included in the explanatory variables. Gender was coded as ‘male’ or ‘female’; age was grouped as ‘less than 30 years’, and ‘equal or more than 30’; marital status was coded as ‘ever married’ or ‘never married’; hospital type was coded as ‘public’ or ‘private’; working department was coded as ‘emergency’, ‘surgery’, and ‘other departments’; and type of employment was coded as ‘contractual’ or ‘permanent’.

### Statistical analysis

We coded and analysed the data from the completed surveys with SPSS for Windows, Version 26. Absolute frequencies and percentages were used to express categorical variables. Basic information like demographics and how they relate to physical violence against doctors was summarised using cross-tabulation.

A Chi-square test was carried out to investigate the association between sociodemographic characteristics and physical violence against doctors. Shapiro–Wilk and Kolmogorov–Smirnov tests were conducted to determine the normality of numeric data about physical violence. As the data were not normally distributed, therefore, non-parametric tests, such as Kruskal–Wallis test statistic or Mann–Whitney *U* tests, were conducted to analyse the association between sociodemographic characteristics and physical violence. Finally, we performed the binary logistic regression model for predicting the physical violence against doctors. For all statistical analyses, the α level (significance) was 0.05.

## Results

Table 1 shows the background characteristics of the chosen covariates and the frequency of physical violence against doctors in Bangladeshi tertiary care hospitals. A total of 50 doctors (12.3%) reported to exposure physical in Bangladeshi tertiary care hospitals in the 12 months prior to the survey. The percentage of physical violence at work against doctors was higher for those who were under 30 years (70%), were male (64%) and had never been married (68%) as well as for those who provided services to the public hospital (78%), permanent employee (66%), and worked in the emergency department (50%). From the Chi-square test we found that age, marital status, and department were highly significant for physical violence against doctors (*p* < 0.001).

**Table 1:** Assessing association between selected covariates and physical violence against doctors in tertiary care hospitals using χ^2^ test (*n* = 406)

Participants’ characteristics	Physical violence		χ^2^	*P* value
	Yes (*n* = 50; 12.3%)	No (*n* = 356; 87.7%)		
	*n* (%)	*n* (%)		
Age in years				
Less than 30	35 (70.0)	118 (33.1)	17.512	< 0.001
Equal or more than 30	15 (30.0)	238 (66.9)		
Gender				
Male	32 (64.0)	175 (49.2)	3.865	0.049
Female	18 (36.0)	181 (50.8)		
Marital status				
Never married	34 (68.0)	106 (29.8)	28.355	< 0.001
Ever married	16 (32.0)	250 (70.2)		
Hospital				
Public	39 (78.0)	222 (62.4)	4.671	0.031
Private	11 (22.0)	134 (37.6)		
Type of work				
Permanent	33 (66.0)	272 (76.4)	2.540	0.081
Contractual	17 (34.0)	84 (23.6)		
Department				
Surgery	13 (26.0)	48 (13.5)	25.892	< 0.001
Emergency	25 (50.0)	67 (18.8)		
Other departments	12 (24.0)	248 (67.7)		

Other departments: Medicine, Orthopedics, ENT, Pediatrics, Intensive Care, Gynae & Obstetrics

Table 2 shows the characteristics of physical violence in tertiary care hospitals against doctors in Bangladesh. Patients’ relatives were recognised as the major perpetrators (72.0%, *n* = 36), followed by the visitors (24.0%, *n* = 12) and patients (4.0%, *n* = 2). Approximately 26% (*n* = 13) of the doctors were injured due to physical assault. After being physically assaulted, 72% (*n* = 36) missed workdays/took sick leave, and 72% (*n* = 36) did not receive support from hospital officials. Moreover, 88% (*n* = 44) of doctors were very worried in their workplace due to physical violence.

**Table 2:** Characteristics of physical violence against doctors in tertiary care hospitals (*n* = 50)

Characteristics of physical violence	Number	Percentage
Perpetrators’ characteristics		
Patients	2	4.0
Relative of patients	36	72.0
Visitors or unknown	12	24.0
Injured caused by perpetrators		
Yes	13	26.0
No	37	74.0
Took a day off from work after being assaulted		
Yes	36	72.0
No	14	28.0
Support or counsel from hospital authorities		
Yes	15	30.0
No	35	70.0
Possibility of preventing this incident		
Yes	48	96.0
No	2	4.0
Worries about current workplace violence		
A little worried	4	8.0
Worried	2	4.0
Very worried	44	88.0

**Fig. 1:**
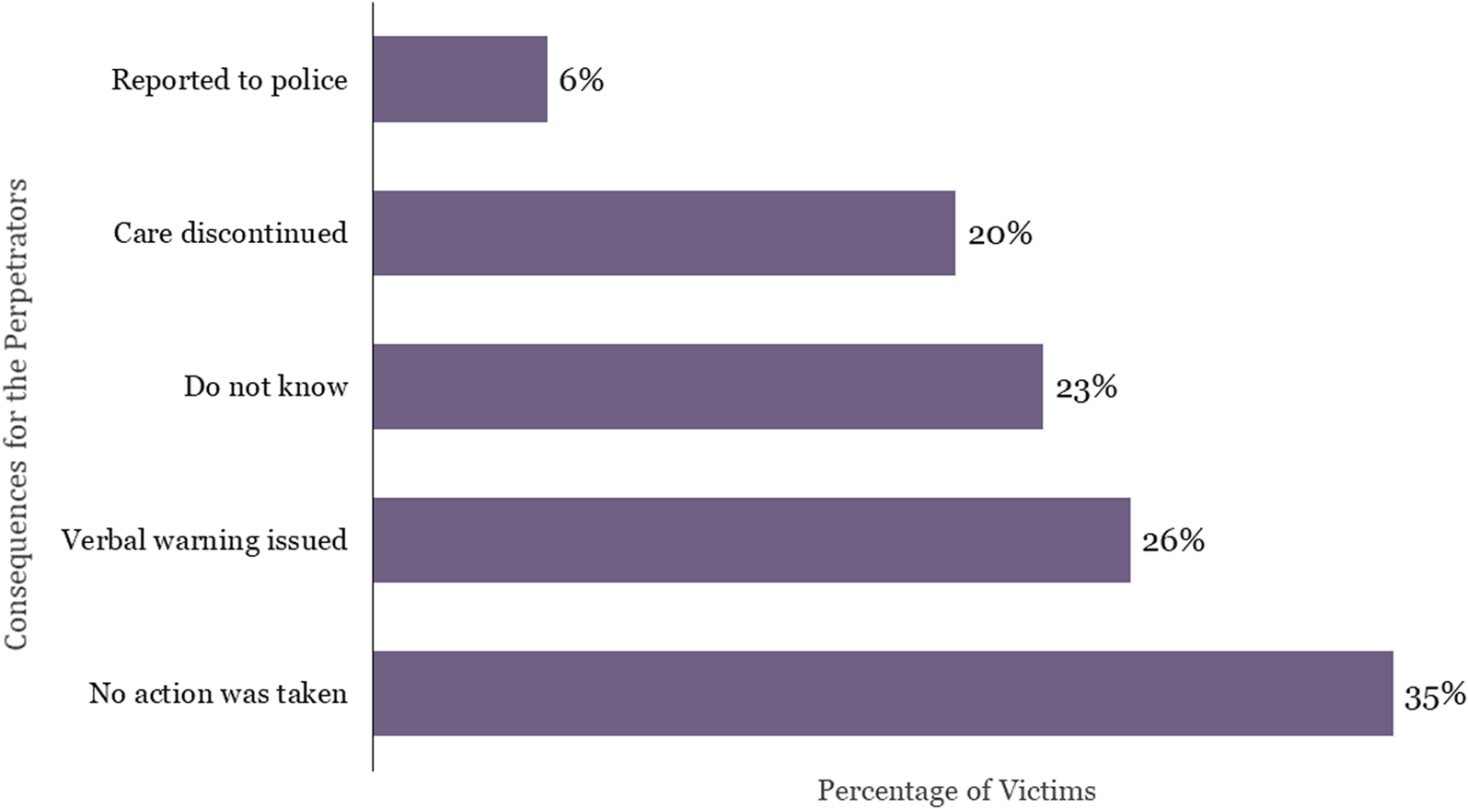
Perpetrators’ consequences according to victims

Regarding the perpetrators’ consequences, almost 35% of victims claimed that no action was taken to investigate the incident, while 26% reported that a verbal warning was issued after the incident, 23% indicated that they had no idea about consequences, 20% claimed that care was immediately discontinued after the incident, and 6. % declared that the violence was reported to the police (Fig. 1).

We employed the binary logistic regression model to determine the risk factors for physical violence against doctors are displayed in Table 3. For both unadjusted and adjusted odds ratios, public hospitals and emergency departments were statistically significant to physical violence against doctors at a 95% confidence interval (see Table 3). Table 3 also presents additional information about the association between physical violence against doctors and various demographic and work-related factors. The adjusted odds ratios (aORs) showed that doctors under the age of 30 years had a higher risk of experiencing physical violence (aORs: 1.324, 95% CI: 0.144 to 4.973) compared to their older counterparts. Male doctors were also more likely to experience physical violence compared to female doctors (aORs: 1.534, 95% CI: 0.761 to 6.096), while never-married doctors had a higher risk of experiencing physical violence than married doctors (aORs: 0.236, 95% CI: 0.029 to 1.786), although this association was not statistically significant. The study also found that doctors working in public hospitals had a significantly higher risk of experiencing physical violence compared to those in private hospitals (aORs: 2.376, 95% CI: 1.210 to 10.080). Additionally, doctors working in emergency departments had a higher likelihood of experiencing physical violence compared to those in surgery departments (aORs: 2.011, 95% CI:1.470 to 9.042).

**Table 3:** Risk factors associated with physical violence against doctors in tertiary care hospital; binary logistic regression model results

Participants’ characteristics	Physical violence (*n* = 50)			
	ORs	95% CI (lower- upper)	aORs	95% CI (lower- upper)
Age in years				
Equal or more than 30 (Ref.)				
Less than 30	1.345	0.247–3.595	1.324	0.144–4.973
Gender				
Female (Ref.)				
Male	1.544	0.894–5.005	1.534	0.761–6.096
Marital status				
Ever married (Ref.)				
Never married	0.250	0.106–0.377	0.236	0.029–1.786
Hospital				
Private (Ref.)				
Public	2.467	1.231–10.943	2.376	1.210–10.080
Type of work				
Permanent (Ref.)				
Contractual	1.668	0.885–3.145	1.674	0.776–3.614
Department				
Surgery (Ref.)				
Emergency	2.045	1.096–9.254	2.011	1.470–9.042
Other departments	0.935	0.044–2.453	0.671	0.025–4.170

Adjusted odds ratio = aORs, unadjusted odds ratio = OR, confidence interval = CI, and reference = Ref.

**Fig. 2:**
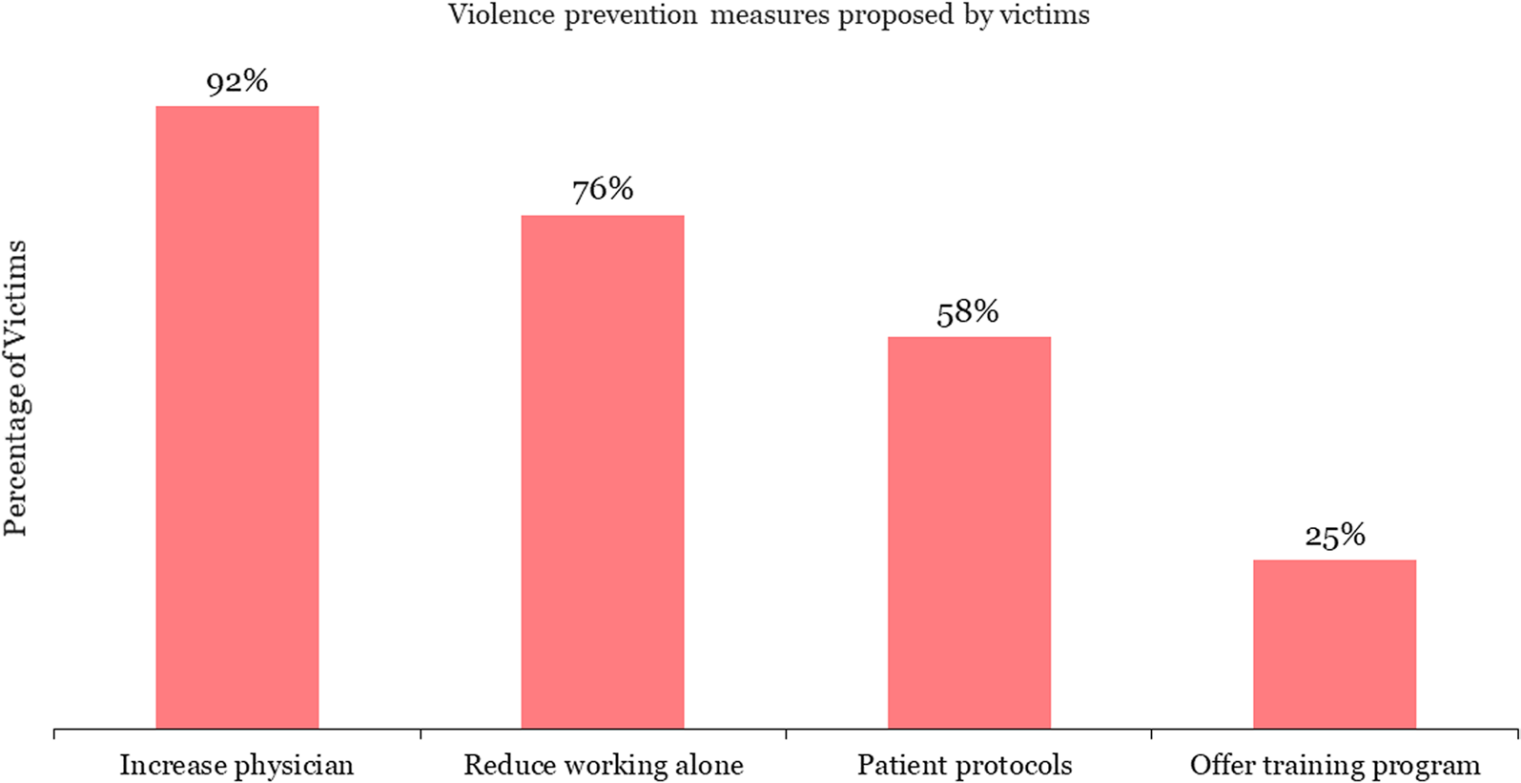
Suggested measures from victims for preventing physical violence in hospitals (*multiple responses)

The recommendations from doctors who were exposed to physical violence for tackling the violence in hospitals are seen in Fig. 2. The victims were requested to make recommendations for ways to avoid a repeat of the violence incident in the hospitals. Out of 50, 46 (92.0%) victims suggested increasing doctors, 38 (76.0%) proposed shortening the time spent working alone, 29 (58.0%) suggested maintaining patient protocols and 25 (50.0%) urged arranging training programmes for tackling hospital violence.

## Discussion

Results from this survey show that physical violence is highly prevalent among doctors in Bangladeshi tertiary care hospitals, particularly males and young doctors working in emergency departments and public hospitals are at high risk. These findings highlight the urgent need to implement a policy focused on safe workplaces for doctors to promote violence prevention in the national health system.

In the present study, 12.3% of physicians experienced physical violence in tertiary care hospitals in the past 12 months before the survey. This percentage is lower to what was previously reported in China (16.0%) [25] and Pakistan (17.7%) [26]. There may be discrepancies among these studies because of the different assessment systems and geographical locations. Moreover, the frequency of physical violence among male doctors was higher than female doctors. This result is in stark agreement with a previous study in Bangladesh [14] and other research findings [6, 14, 27, 28] from different parts of the world. The reason may lie in the cultural norms of avoiding observing violence against women. There is also the possibility that women are less likely to report violent incidents because they are afraid of negative consequences or self-image, or women have less opportunity to be open about their situation in their workplace [3, 16]. However, reporting is an essential precondition for an effective response [3]. This may also be attributed to the urgency of the need for attention and the typical absence of reporting systems in Bangladeshi hospitals [14]. Therefore, a proper reporting system for doctors, patients, or visitors should be introduced in hospitals, and meticulous records should be kept to monitor the situation for timely intervention.

The present study found that young doctors had a higher risk of experiencing physical violence compared to their older counterparts. Similar findings were found in the previous studies [6, 13, 14]. A possible explanation could be that senior doctors have better communication skills and are more professional at handling agitated and nervous patients and managing potentially problematic situations compared to younger doctors. Another reason could be that younger doctors are usually the most available and more accessible to patients and visitors than senior doctors, hence most likely to be the first victims of workplace violence [19]. Therefore, the authors suggest that the authorities must introduce staff training in skills, cultural diversity, interpersonal communication, conflict management, and teamwork to prevent hospital violence in Bangladesh. It is pertinent to mention that the above recommendations have been encouraged in several studies to eliminate hospital violence or help doctors better manage in many countries [3, 32]. Further, the Bangladesh Medical and Dental Council can integrate communication skills into medical education to ensure that future doctors develop essential knowledge on managing patients.

Similar to previous Bangladeshi studies, the result of this study shows that doctors working in public hospitals had a significantly higher risk of experiencing physical violence than in private hospitals [14, 23]. This finding is supported by previous international studies that found doctors were at high risk of exposing physical violence in public hospitals [6, 10–12]. In Bangladesh, a lack of resources, poor logistical planning, and unavailability of adequate health workers, particularly doctors in primary and secondary hospitals lead to patient being referred to tertiary care hospitals [19, 21, 23]. Moreover, the availability of free or low-cost treatment in Bangladeshi public hospitals attracts people daily, pushing doctors to treat an overwhelming number of patients [30]. It is pertinent to mention that our study hospitals are medical college hospitals where modern medical technologies and specialised doctors are available [21]. Among our study hospitals, specifically in public hospitals, the treatments are low-cost and, in some cases, free [30]. As a result, patients tend to visit these health facilities for better health care services, affordability, and low cost, which causes an overload of patients. From another point of view, this is probable, as the concentration of doctors and, thereby, patients are much higher in urban areas in absolute numbers [3, 7]; also, our study hospitals are large and located in urban areas, which get to handle an abundance of complicated cases with higher morbidity and mortality. Thus, this situation can lead doctors to experience more stress in managing diverse patients and conflict and possibly cause violence. This is attributed to the urgency of the need for attention and unorganised patient protocols in tertiary care hospitals in Bangladesh. Further research is needed to determine how these variables relate to violence in Bangladeshi hospitals.

According to this data, physical violence incidents were most common in the emergency departments. This finding follows a recent study result found more than half of the violent incidents took place in the emergency departments in Bangladesh [14]. Similarly, several studies from different parts of the world emphasised the higher rate of violent incidents in emergency departments [6, 13, 16, 31]. This department is the doorway to the inpatient departments, which is why it is the first department to deal with critical patients, followed by the inpatient departments [13]. The patients often die either in an emergency room or inpatient ward despite the best efforts of the doctors and other health workers. However, in most cases, patients' relatives or visitors typically blame doctors for all defects, which in turn exposes them to aggression, leading to violent incidents [3, 13, 31]. Thus, this department is deemed particularly risky for physical violence, where managing heterogeneous patients of various ages, mentalities, and disease severity is always challenging [13]. Future research focusing on this setting could potentially identify the key factors responsible for violence in each hospital department or ward.

Our study demonstrates how physicians are affected by physical violence differently depending on their situations and the perpetrators. This study found that two-thirds of the perpetrators of violence were relatives of the patients. Similarly, existing literature in this country [14, 19, 21] and elsewhere [2, 10, 31] indicated that patients’ relatives were primarily responsible for committing violence in hospitals. This may be due to a lack of empathy amongst relatives of patients for doctors. For example, when individuals are exposed to critical health conditions and wait for long times until seen by a doctor, and unmet needs or expectations such as high-level medical services even for minor, self-limiting conditions are not met; as a result, they and their relatives may experience high levels of stress, anger, and frustration, which may manifest as violence against physicians [32]. Consequently, their negative emotions are probably vented through extreme violence towards doctors as if they even resort to harming doctors violently. Thus, doctor–patient contradictions are created and escalate, leading to further violence. On the other hand, a lack of humanity in treating patients and a lack of respect for patients’ dignity may account for the aggression perpetrated by patients and relatives [3, 32]. Therefore, there is a need to determine the reason behind poor doctor–patient relations, particularly in hospital settings in Bangladesh. Moreover, the authors suggest that Bangladeshi hospitals need to follow adequate patient protocols, display a code of conduct, and have better control over visitors and staff movement to mitigate hospital violence in agreement with other research recommendations [3, 7].

According to our study data, around a quarter of victims claimed they had no idea of the consequences of the perpetrators’ actions and the same numbers of victims discontinued their regular duty or care in the hospitals due to being exposed to physical violence. This finding is in line with a previous study conducted among healthcare workers in Bangladesh [14]. Thus, their discontinued care may undermine equal health care and healthcare delivery access which indicate the defects of specific medical system. There are many adverse outcomes associated with violence in hospitals. For example, exposure to violence significantly affects doctors’ psychological stress levels, sleep quality, and health [3, 7, 33, 34]. It was also found that participants who were subjected to physical violence and had been exposed to it were more likely to experience depression and anxiety symptoms compared to those who had not [3, 33, 36]. Here, we observed that two-thirds of the doctors were very worried about violence in the current workplace. If physical violence against doctors becomes frequent, it would tremendously affect them and ultimately have a ripple effect on the country’s health system.

Our findings highlight that employee increases, specifically among doctors and reduce working alone were the most widely implemented measure among the recommendations from victims to tackle violence in the hospital. The Ministry of Health and Family Welfare reports that Bangladesh has only 6 doctors, nurses, and midwives per 10,000 people, which second lowest in South Asia [37]. According to the World Bank collection of development indicators, the number of physicians (per 1000 people) in Bangladesh was reported at 0.6367 in 2019 [38]. This is a very incompatible ratio, indicating a lack of human resources for health. Notably, this staff shortage prevents doctors from meeting patients’ demands linked to poorer health care system delivery in Bangladesh [21]. This leads to overwork, stress, and dissatisfaction among doctors, resulting in poorer delivery of care [1, 5, 16]. It is undeniable that these issues pose a threat to doctors and other health workers and result in violence at the workplace [1, 3, 7, 33, 35]. Therefore, there is an urgent need to find adequate solutions to this issue since further violence in hospitals will likely worsen the impending burnout and shortage crisis that doctors are currently experiencing in Bangladesh.

The findings of this study have some policy implications. First, the high incidence of physical violence in tertiary care hospitals exhibits the country’s lack of ability of a health system to resolve the issue. Concurrently, it highlights the need for an urgent call for policies and measures for zero tolerance of violence against Bangladeshi doctors. For example, a legislation identical to the Indian government-approved law to protect from workplace violence in 2020 which recognised any violence against healthcare personnel as a cognisable and non-bailable offence [38] or a national ordinance similar to what Nepal did with its ordinance in 2022 [40]. Second, to ensure equitable and inclusive access to quality health care, policymakers must invest in human resources for health, particularly doctors in the country’s health facilities based on population, to achieve universal health coverage and SDG target 3 [35]. The WHO Global Strategy on Human Resources for Health 2030 [41] stipulates that the health workforce is essential to meet SDGs [22]. Similarly, a recent report has highlighted that human resources for health is one of the major setbacks to implementing the SDGs in Bangladesh [21]. Although the WHO reported that from 2007 to 2020, Bangladesh experienced an increase of 12% in the presence and accessibility of qualified health workers, this increase is not enough to meet the demands of the country’s population [42]. Therefore, the lack of human resources for health challenges and gaps needs to be addressed promptly, not only to tackle workplace violence but also to ensure safe and accessible health care to all. Third, our findings also suggested that it is crucial to have adequate numbers of skills (e.g., training, workshop) for doctors to cope with violence and mental stress abstain from absenteeism and burn out [14, 19]. Hence, prevention measures might include developing education and training programmes to help doctors better manage workplace violence. Finally, it needs to be addressed at the policy level to understand the reasons for physical violence and the factors at stake to evolve effective preventive policies and actions. Therefore, the authors strongly recommend drawing up an analysis of the grounds and causes of physical violence in primary to tertiary-level hospitals. This understanding is often overlooked [14, 19, 23], but regardless of the situation, it is crucial for the country’s health system.

The current study has a number of strengths. To our knowledge, this is the first published article to estimate the prevalence of physical violence among doctors in Bangladesh. This survey examined doctors from the country’s a number of largest fully functional randomly selected public and private hospitals helped reducing selection bias. In addition, physical aspects were included in the questionnaires and a definition of physical violence was provided following the WHO guidelines. This survey also has some limitations. First, the findings of this study cannot be generalised to all-care level hospitals since it did not include doctors from primary and secondary care hospitals who are also at high risk of physical violence. Therefore, a large-scale study that includes patients and doctors from all hospital levels is needed to capture a fuller extent of hospital-based violence accurately. Second, this study was limited to doctors while other health workers are (e.g., nurse, paramedics, staffs) more likely to experience physical violence. Third, we did not assess the severity of physical violence and identify gender of the perpetrators. Finally, it was a cross-sectional study and the data were collected retrospectively which could be influenced by recall bias.

## Conclusion

Physical violence is highly prevalent among doctors in Bangladeshi tertiary care hospitals, particularly males and young doctors working in emergency departments and public hospitals are at high risk. There is an urgent need for governments and stakeholders to prioritise increasing employees, particularly doctors, based on people’s needs, reduce duration of working alone and maintain patient protocols in hospitals. Concurrently, a proper reporting system and a counselling centre should be executed in the hospitals. The policymakers must commit to establish hospitals as safe workplaces where violence is not tolerated and adopt legislation to protect doctors from violent incidents. Future large-scale studies are needed to investigate the causes and possible solutions for tackling physical violence against doctors in Bangladeshi hospitals.

## Supplementary Information

**Additional file 1. **Survey Questionnaire for the prevalence of determinants of physical violence against doctors in Bangladeshi tertiary care hospitals.

**Additional file 2. **Flow diagram of survey procedures.

## Data Availability

The datasets used and/or analysed during the current study are available from the corresponding author on reasonable request.

## Abbreviations

ENT: Eye, Nose and Tongue
SDG: Sustainable Development Goal
WHO: World Health Organization
ILO: International Labour Organization
