# Supplementary material for: Prevalence and determinants of physical violence against doctors in Bangladeshi tertiary care hospitals
Source: Hum Resour Health. 2023 Mar 28;21:26. doi: 10.1186/s12960-023-00811-x (PMC10045192; doi:10.1186/s12960-023-00811-x)
Supplement: Supplementary file 1 — Additional file 1. Survey Questionnaire for the prevalence of determinants of physical violence against doctors in Bangladeshi tertiary care hospitals. [file 12960_2023_811_MOESM1_ESM.pdf]

Prevalence and determinants of physical violence against doctors in Bangladeshi tertiary care hospitals

**Personal information**		
**P1**	Gender	☐ Male ☐ Female
**P2**	Age (year)	
**P3**	Marital status	☐ never married ☐ married
**P4**	Employment	☐ public ☐ private
**P5**	Job types	☐ permanent ☐ contractual
**P6**	Working department	☐ general medicine ☐ general surgery<br>☐ emergency ☐ intensive care<br>☐ management ☐ pediatrics<br>☐ gynae & obs ☐ orthopedics<br>☐ dermatology ☐ others, please specify_____
**Physical violence related information**<br>**Note**: *Physical violence refers to pinching, pushing, shoving, kicking, raped attempted.*		
**PV1**	**In the last 12 months**, have you been a victim of physical violence in your workplace?	☐ Yes<br>☐ No; *if No, stop here please.*
**PV2**	Who was aggressive towards you?	☐ patient ☐ relatives of patient<br>☐ staff member ☐ visitors/unknown<br>☐ other………
**PV3**	Were you injured as a result of the violent incident?	☐ yes ☐ no
**PV4**	Did you have to take time off from work after being attacked?	☐ yes ☐ no
**PV5**	Do you think the incident could have been prevented?	☐ yes ☐ no
**PV6**	What were the consequences for the attacker?	☐ None ☐ verbal warning issued<br>☐ care discontinued ☐ reported to police<br>☐ other:______________ ☐ don't know
**PV7**	Did your employer or supervisor offer to provide you any support?	☐ yes ☐ no
**PV8**	How worried are you about violence in your current workplace?	☐ not at all ☐ a little ☐ worried<br>☐ very worried
**Suggestion for tackling physical violence**		
**S1**	To what extent do you think these measures would be helpful in your work setting?	☐ Increase doctors<br>☐ Patient protocols<br>☐ Reduced periods of working alone<br>☐ Training for tackling violence<br>☐ Others, specify please: …………………

Thank you for your contribution
