# Supplementary figures and images for: Prevalence and determinants of physical violence against doctors in Bangladeshi tertiary care hospitals
Source: Hum Resour Health. 2023 Mar 28;21:26. doi: 10.1186/s12960-023-00811-x (PMC10045192; doi:10.1186/s12960-023-00811-x)

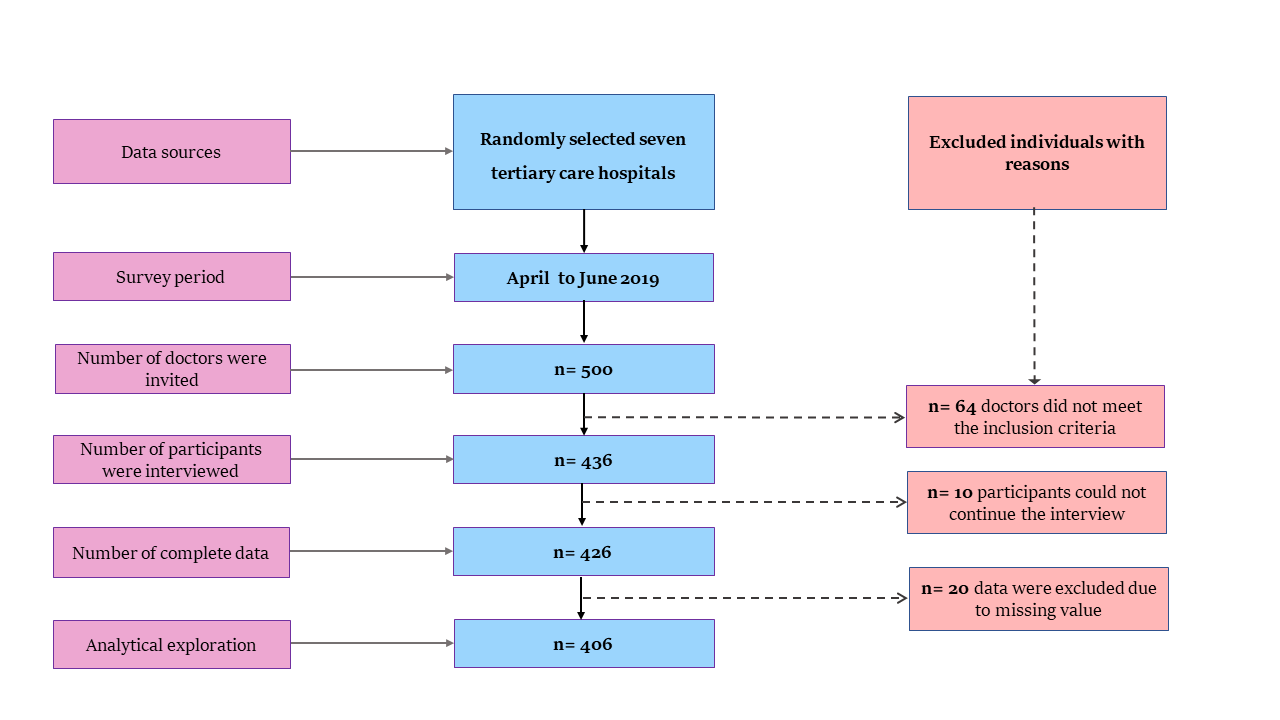

Supplement: Supplementary file 2 — Additional file 2. Flow diagram of survey procedures. [file 12960_2023_811_MOESM2_ESM.png]
